# Redundant and Diversified Roles Among Selected *Arabidopsis thaliana* EXO70 Paralogs During Biotic Stress Responses

**DOI:** 10.3389/fpls.2020.00960

**Published:** 2020-06-26

**Authors:** Tamara Pečenková, Andrea Potocká, Martin Potocký, Jitka Ortmannová, Matěj Drs, Edita Janková Drdová, Přemysl Pejchar, Lukáš Synek, Hana Soukupová, Viktor Žárský, Fatima Cvrčková

**Affiliations:** ^1^ Institute of Experimental Botany, CAS, Prague, Czechia; ^2^ Department of Experimental Plant Biology, Faculty of Science, Charles University, Prague, Czechia

**Keywords:** exocyst, EXO70, *Arabidopsis thaliana*, redundancy, gene expression, lipid binding, biotic stress, root hairs

## Abstract

The heterooctameric vesicle-tethering complex exocyst is important for plant development, growth, and immunity. Multiple paralogs exist for most subunits of this complex; especially the membrane-interacting subunit EXO70 underwent extensive amplification in land plants, suggesting functional specialization. Despite this specialization, most Arabidopsis *exo70* mutants are viable and free of developmental defects, probably as a consequence of redundancy among isoforms. Our *in silico* data-mining and modeling analysis, corroborated by transcriptomic experiments, pinpointed several EXO70 paralogs to be involved in plant biotic interactions. We therefore tested corresponding single and selected double mutant combinations (for paralogs EXO70A1, B1, B2, H1, E1, and F1) in their two biologically distinct responses to *Pseudomonas syringae,* root hair growth stimulation and general plant susceptibility. A shift in defense responses toward either increased or decreased sensitivity was found in several double mutants compared to wild type plants or corresponding single mutants, strongly indicating both additive and compensatory effects of *exo70* mutations. In addition, our experiments confirm the lipid-binding capacity of selected EXO70s, however, without the clear relatedness to predicted C-terminal lipid-binding motifs. Our analysis uncovers that there is less of functional redundancy among isoforms than we could suppose from whole sequence phylogeny and that even paralogs with overlapping expression pattern and similar membrane-binding capacity appear to have exclusive roles in plant development and biotic interactions.

## Introduction

The exocyst complex is evolutionarily conserved across eukaryotes and mutations impairing its function are often lethal, suggesting its important biological role (Koumandou et al., 2007; Martin-Urdiroz et al., 2016). Initial knowledge of the exocyst comes especially from studies in yeast, where this heterooctameric protein complex regulates exocytosis by mediating the physical tethering of secretory vesicles to the target plasma membrane (TerBush et al., 1996; Hsu et al., 2004). A distinct role in the process of tethering has been ascribed to each of the eight exocyst subunits. The SEC10 and SEC15 subunits mediate interaction of the complex with a vesicle *via* Rab GTPases (Roth et al., 1998; Guo et al., 1999). The EXO70 and SEC3 subunits direct the complex to the target membrane through an interaction with membrane phosphatidylinositol 4,5-bisphosphate (PIP2) (Boyd et al., 2004; He et al., 2007; Liu et al., 2007; Pleskot et al., 2015). The SEC6 subunit has been found to provide for an interaction with the SNARE complex but also to form the inner core of the complex with SEC8 (Dubuke et al., 2015; Picco et al., 2017), while subunits EXO84 and SEC5 mediate activation by Ral type GTPases (Moskalenko et al., 2002). The performance of the tethering function also requires an allosteric regulation of the EXO70 by the RHO GTPases (Wu et al., 2010; Rossi et al., 2020).

In land plants, several exocyst subunits underwent gene amplification, allowing formation of alternative complexes with at least partially distinct roles in the growth, development, and biotic interactions. In particular, the EXO70 subunit has evolved into a large gene family that *e.g.* in the genome of the model plant *Arabidopsis thaliana* consists of 23 EXO70 paralogs that can be classified into three subfamilies/clades and further into eight groups A-H (A in subfamily 1, B, C, D, E, F, H in subfamily 2 and G in subfamily 3; Elias et al., 2003; Synek et al., 2006; Chong et al., 2010; Cvrčková et al., 2012; Rawat et al., 2017; Žárský et al., 2020). Thus, the Arabidopsis exocyst can consist of SEC6, SEC8, one of 23 EXO70 isoforms, and one of two isoforms of SEC3, SEC5, SEC10, SEC15, and EXO84 each, resulting in 736 theoretically possible combinations that may act in various endomembrane compartments and plasma membrane domains, cells and tissues, developmental stages, and environmental situations. Even if the number of really occurring variants is substantially lower due to tissue-specific expression of some subunits isoforms, there are still numerous exocyst variants that must be coordinated in space and time to ensure proper functioning of the plant cell and organism (Žárský et al., 2013).

The multiplicity of the plant exocyst complex functions has been well documented. Distinct exocyst variants are involved in auxin transport, root and hypocotyl epidermal cell elongation, cytokinesis, pollen tube growth, seed coat formation, defense against pathogens, xylem differentiation, and leaf trichome development (Cole et al., 2005; Synek et al., 2006; Hála et al., 2008; Kulich et al., 2015; Sekereš et al., 2017; Vukašinović et al., 2017; Janková Drdová et al., 2019). On the subcellular level, exocyst participates in plasma membrane protein recycling, cytokinesis, autophagic targeting to the vacuole, and deposition of cell wall material, especially callose, to the pathogen attack site (Fendrych et al., 2010; Pecenková et al., 2011; Drdová et al., 2013; Žárský et al., 2013; Kulich et al., 2018; rev. in Pečenková et al., 2017b). It is well documented that several versions of the exocyst function simultaneously within the same cell (Sekereš et al., 2017).

As a consequence of the subunits’ genes multiplication, especially in the case of EXO70, single gene knock-out mutations are unlikely to have dramatic phenotypes because of anticipated functional redundancy among the numerous paralogous genes, albeit there are a couple of exceptions. Loss of the main housekeeping variant EXO70A1 causes pleiotropic growth and developmental defects and sterility (Synek et al., 2006), and the double *exo70C1exo70C2* mutant cannot be produced due to at least one of these subunits, highly similar in terms of both sequence and gene expression pattern, being necessary for pollen tube tip growth (Synek et al., 2017).

While *A. thaliana* EXO70 isoforms are generally highly conserved, they are more diverged at their N- and C-termini, including a hypothetical lipid/PI(4,5)P2-binding C-terminal motif (Žárský et al., 2009). This variability indicates a possibility that this region contains a clue for the isoforms’ functional specificity due to a differential lipid-binding capacity. During the various abiotic and biotic stress reactions, phospholipases are activated affecting the content of phosphatidyl inositol phosphate (PIP) species and phosphatidic acid (PA) that further function as both signaling and protein recruiting components of stress responses (review in Laxalt and Munnik, 2002; Wang, 2004). Therefore, the plant cell’s need for multiple EXO70s may reflect the requirement for trafficking toward the target membranes with different lipid composition.

In order to explore the extent of specificity and redundancy among Arabidopsis EXO70 paralogs, we analyzed available transcriptome data with focus on response of individual paralogs to biotic stresses. Based on the expression patterns’ overlaps, we generated several double mutants, aiming to achieve phenotypic deviations enhanced in comparison to corresponding single mutants. Indeed, our two approaches exploring the sensitivity to pathogenic bacteria *Pseudomonas* sp., *i.e.* a flooding assay (Ishiga et al., 2011) and a root hair growth stimulation assay (Pečenková et al., 2017a), revealed either aggravation or suppression of defense-related phenotypes for some of the mutants’ combinations. For several selected isoforms we also characterized both *in silico* and experimentally their lipid binding affinities, assuming that the lipid-binding capacity may hold one of the keys to functional specificity. Our results are documenting on-going evolutionary specialization of isoforms that may both overlap and compete in their functions.

## Materials and Methods

### Plant Material and Growth Conditions

The following previously published Arabidopsis mutant lines were used in this study: *exo70A1-2* (SALK_135462, Synek et al., 2006), *exo70B1-1* (GABI_114C03, Kulich et al., 2013), *exo70B2-2* (SAIL_339-D07, Pecenková et al., 2011), *exo70E1* (SALK_084145, Redditt et al., 2019; primers for genotyping in **Supplementary Table 1**) *exo70F1* (SALK_036927, Synek et al., 2006), and *exo70H1* (SALK_042456, Pecenková et al., 2011). The following double mutants were generated by crossing: *exo70A1-2exo70B1-2* (further on *exo70A1exo70B1*), *exo70A1-2exo70B2-2* (further on *exo70A1exo70B2*), *exo70B1-1exo70B2-2* (*exo70B1exo70B2*), *exo70B2-2exo70H1* (*exo70B2exo70H1*), and *exo70E1exo70F1*, and the triple mutant *exo70A1-2exo70B2-2exo70H1 (exo70A1exo70B2exo70H1*). The wild type (WT) Col-0 or out-crossed sister WT lines were used as controls to single and double mutant lines in pathogen sensitivity experiments. Primers used for the genotyping were the same as used in the above-cited original studies.

For seedlings and plants’ cultivation, seeds were surface sterilized (3 min in 70% ethanol, 2 × 5 min in 10% commercial bleach, rinsed three times in sterile distilled water) and stratified for 2–3 days at 4°C. Seeds were then germinated and grown on vertical 1/2 × MS agar plates (half-strength Murashige and Skoog salts, Duchefa Biochemie, supplemented with 1% sucrose, vitamin mixture, and 1.6% plant agar, Duchefa Biochemie) at 21°C and 16 h of light per day for seven days.

### Gene Expression Analysis

Published microarray transcriptome data were analyzed using the Genevestigator gene expression tool (Hruz et al., 2008). We only included Affymetrix ATH1 Arabidopsis arrays which provide more extended coverage of conditions in comparison to *e.g.* RNASeq. The expression data for each EXO70 isoform gene involved in various developmental stages, anatomic parts, and under different biotic stress conditions were data-mined using Genevestigator tools. For the developmental course of gene expression, average values are presented, typically from several hundreds to 2,000 samples, with exception of siliques and senescent leaves, where the number of samples was less than 100. Expression data from tissues and cell types and from biotic stress treatments are presented as heat maps either as different shades (absolute expression levels in anatomical parts) or in different colors (representing relative expression compared to control; green—downregulation, red—upregulation). The color scales with heat maps are given in log2 ratio values. Hierarchical clustering analysis was performed with Hierarchical Clustering Tool of Genevestigator using Pearson correlation for all or selection of genes for relevant experiments excluding combinations of simultaneous treatments and stresses and experiments which include mutant lines expression data.

Expression data from our own study comparing gene expression in *exo70A1* mutants and isogenic WT seedlings have been obtained and analyzed as reported previously (Hála et al., 2010); the complete data are available as GEO data set GSE18986 (https://www.ncbi.nlm.nih.gov/geo/).

### Assay of Bacterial Stimulation of Root Hair Growth

For the root hair growth stimulation assay by pathogenic bacteria, the experiments were performed as described in (Pečenková et al., 2017a). Briefly, inoculation was performed with *P. syringae* pv. *maculicola* ES4326 (Psm) as follows: cultures from freshly inoculated plates (Luria–Bertani medium with 25 mg/l of rifampicin and streptomycin) were used to prepare liquid overnight culture (40 ml; with incubation at 28°C on an orbital shaker at 130 rpm). Bacteria were centrifuged at 1,500 g for 10 min and the pellet was resuspended in sterile distilled water (dH_2_O) and diluted to an OD_600_ of 0.3 (10^8^ CFU/ml). Approximately 10 μl droplets were applied to cover the root tips within the elongation and differentiation zone. As a mock control, dH_2_O was applied in the same manner and, 48 h later, the root hair growth was inspected. Root hair lengths were analyzed using AnalySIS (Soft Imaging System GmbH, Germany) or ImageJ (Schindelin et al., 2015) software. The numerical data obtained (sample sizes for each of the lines are presented in **Supplementary Table 2**) were processed using Microsoft Excel. Experimental values were analyzed in R using Welch’s corrected one-way analysis of variance (ANOVA), and the pairwise multiple comparisons were done with Games–Howell *post hoc* test at P < 0.05.

### Flooding Assay

Flooding assay was performed with modifications according to Ishiga et al. (Ishiga et al., 2011; Kalachova et al., 2019). Briefly, we used P. syringae pv. tomato DC3000 mutant strain hrpH− [Pst hrpH−; donated by Chris Staiger, West Lafayette, USA; (Yuan and He, 1996)] in OD = 0, 05, diluted in water with Silwet (0.0025%), for flooding of 2-week-old plants for 2 *min*. Suspension was decanted from plates and seedlings incubated for 24 *h* on 12/12 *h* (day/night). After the incubation, in order to quantify bacterial populations, plants (three to four seedlings in one sample, three samples for each line, usually two to six replications, for WT line which was used as a reference among experiments 12 replications; **Supplementary Table 2**) were weighted, surface sterilized by 70% ethanol and homogenized. Series of dilutions were plated onto LB with rifampicin (25 µg/ml) and chloramphenicol (34 µg/ml) and left for approximately 30 *h* until the colonies become visible and countable. The number of colonies was always normalized to corresponding seedling weights, usually resulting in the range of 10^5^–10^6^ CFU/mg fresh weight. To account for experiment-to-experiment variation, colony forming unit (CFU) values for various mutants in individual experiments were normalized to the CFU of control plants and expressed as a sensitivity fold change; subsequently all data were compared together, using ggplot2 and agricolae packages in R. Kruskal–Wallis and Dunn*’*s *post hoc* tests with the Benjamini–Hochberg correction to test for significant differences at P < 0.05.

### Bioinformatic Analysis of Lipid Binding Motifs

An alignment of *Arabidopsis thaliana* EXO70 sequences has been generated in our previous phylogenetic study (Cvrčková et al., 2012) and is available there, including a full list of sequences. Paralogs of *A. thaliana* EXO70s found to exhibit significant expression in biotic stress have been visually examined for differences between the two Arabidopsis species in the region corresponding to the putative C-terminal lipid-binding site (Kalachova et al., 2019), as well as for conservation of individual positions, with the aid of the BioEdit visualization tool (Hall, 1999).

### Structural Homology Modeling

3D models of near full-length Arabidopsis EXO70B1 (aa 57-618) and EXO70B2 (aa 63-596) were predicted using mouse Exo70 (PDB code 2PFT) and partial Arabidopsis EXO70A1 (PDB code 4RL5) structures as templates, using Modeller 9v8 software (Webb and Sali, 2016). A structure–sequence multiple alignment of 231 EXO70 sequences from multiple eukaryotic lineages was used as a starting point for the prediction. 100 generated models were evaluated with internal Modeller ranking (DOPE-HR, molpdf and Z), and 10 best models were further evaluated using Prosa (Wiederstein and Sippl, 2007) and WhatIf (Hekkelman et al., 2010) algorithms. The best models scored comparable to or even better than the experimental template structures. Electrostatic potential map of the EXO70B1 and EXO70B2 models was calculated using Poisson–Boltzmann equation in the APBS program (Baker et al., 2001). Images were prepared using Pymol and Inkscape software packages (Yuan et al., 2016).

### Cloning Procedures

Constructs in the pGEX4T-2 vector encoding C termini of GST-EXO70B1 (GST-EXO70B1-ct) and GST-EXO70B2 (GST : EXO70B2-ct) were cloned from PCR products obtained using primers shown in **Supplementary Table 1**. Full length constructs of EXO70B1, B2, and H1 were cloned into pTNT vectors providing N-terminal fusion with the HA epitope under the control of the SP6 promoter. List of primers used for cloning is shown in **Supplementary Table 1**. Already existing clones (Pecenková et al., 2011; Kulich et al., 2013) were used as templates for PCR.

### Bacterial Expression and Purification of Proteins

Constructs in the pGEX4T-2 vector encoding C termini of GST-EXO70B1 (GST-EXO70B1-ct) and GST-EXO70B2 (GST-EXO70B2-ct) were used for the transformation of *Escherichia coli* ArcticExpress (DE3) RIL cells with Codon Plus technology (Stratagene). Bacterial culture was grown in LB medium (200 ml) supplemented with ampicillin (50 mg/l) at 37°C until OD_600_ reached 0.5–0.8, then the protein expression was induced by 1 mM isopropyl *β*-D-thiogalactoside. Cells were harvested by centrifugation and resuspended in 5 ml of the lysis buffer (25 mM Tris, 250 mM NaCl, 5 mM beta-mercaptoethanol, protease inhibitors cocktail (Roche), pH 8.0). Bacterial cells were disrupted by sonication, and lysates were cleared by centrifugation. Soluble fractions were loaded on a glutathione-agarose column (Sigma). After two washes with 10 ml of the lysis buffer, the GST-EXO70B1-ct and GST-EXO70B2-ct proteins were eluted by 0.25 ml of 1 M Tris-HCl (pH 8.8) supplemented with 30 mM glutathione.

### Protein Expression In Vitro

For the expression of full versions of proteins EXO70B1and EXO70B2, the TnT^®^ SP6 High-Yield Wheat Germ Protein Expression System (Promega) was used according to the manufacturer’s instructions. Briefly, plasmid DNA with cloned EXO70s genes (2 μg) was mixed with an optimized wheat germ extract containing all the components (tRNA, ribosomes, amino acids, polymerase, and translation initiation, elongation and termination factors) necessary for protein synthesis in a reaction volume of 50 μl and incubated for 60–90 minutes at 25°C. The aliquots of expression reactions were verified by Western blots using anti-HA antibody (Thermo Fisher Scientific). Expressed proteins were used directly for lipid overlay.

### Protein–Lipid Binding Assays

Protein***–***lipid overlay assays with PIP and lipid strips (P-6001 and P-6002 respectively, Echelon Biosciences) were performed according to the manufacturer’s instructions. Briefly, strips were first blocked with 3% fatty acid-free BSA in PBS (3 ml, 10 mM phosphate, and 150 mM NaCl, pH 7.4) for 1 h and incubated 2 h at room temperature with blocking buffer containing 0.5 μg/ml for each of GST-EXO70B1-ct, GST-EXO70B2-ct, HA-EXO70B1, and HA-EXO70B2. The strips were washed 3× with 3 ml of PBS with 0.1% Tween. To detect the proteins, an anti-GST (Echelon Biosciences, dilution 1/2000) or anti-HA mouse monoclonal antibody (Thermo Fisher Scientific, dilution 1/1,000 dilution) was used. Subsequently, chemiluminescence detection (ECL, Amersham) of the secondary anti-mouse antibody (Promega) conjugated with horse radish peroxidase was used for identification of positive interactions. The signal was documented using Bio-Rad documentation system.

## Results

### Gene Expression of EXO70 Paralogs

**Figure 1:**
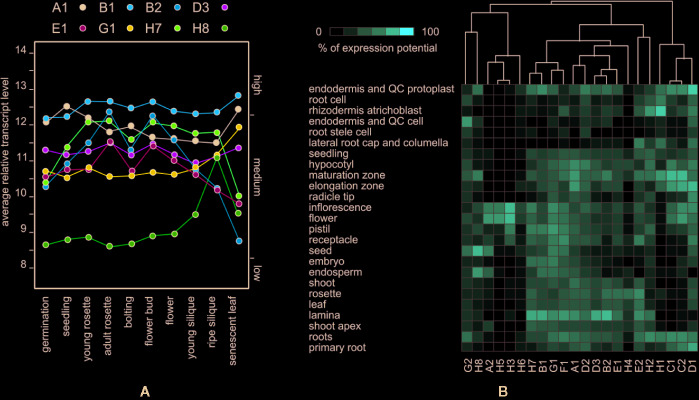
**(A)** Developmental distribution of expression levels of the eight EXO70 isoforms that exhibit a high transcript level at least at one developmental stage. **(B)** Hierarchical clustering of EXO70 isoforms according to expression levels in a selection of anatomical parts. Gene names are abbreviated (A1 = EXO70A1 *etc.*); for standard locus identifiers see (Cvrčková et al., 2012).

We first examined to what extent are the evolutionary relationships between EXO70 paralogs mirrored in their gene expression patterns. All but one of the 23 EXO70 isoforms (except EXO70H6) are covered by the standard Affymetrix ATH1 Arabidopsis arrays. We used Genevestigator (Hruz et al., 2008) to compare the expression of these genes during Arabidopsis development and under varying environmental conditions. Unlike the core exocyst subunits (*e.g.* SEC6 or SEC8) with typically stable expression (Cole et al., 2005), most EXO70 paralogs exhibited low and fluctuating transcript levels during development, and only eight paralogs reached high transcript levels comparable to the core subunits at least at one developmental stage (**Figure 1A**). Only three isoforms, EXO70A1, B1, and D3, maintain stable transcript levels (high in the case of A1 and B1 and intermediate for D3) while EXO70B2, E1, and H7 exhibit varying/variable expression levels. Isoforms G1 and H8 seem to be upregulated in senescent organs and mature siliques, respectively.

The expression of several EXO70 paralogs largely varies across tissues and cell types as reported earlier (Cole et al., 2005; Sekereš et al., 2017; Synek et al., 2017). For example, in the root maturation zone, as well as in root hairs, we found that isoforms EXO70 C1, C2, D1, H1, B2, E1, H7, H2, and F1 are the most abundantly expressed, and a lower level of A1 transcript is present, while D1 and E2 have a transcript level peak in guard cells and B2, H7, and B1 in the leaf mesophyll (**Figure 1B**, **Supplementary Figure 1**).

**Figure 2:**
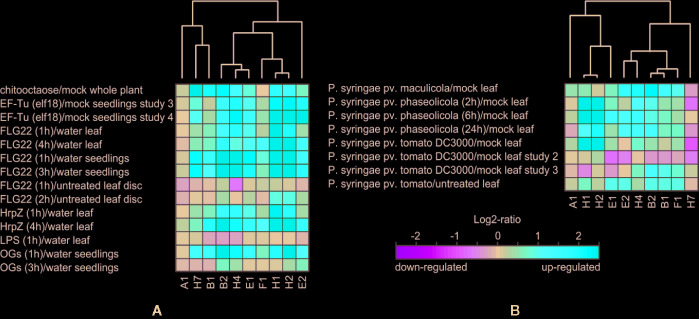
**(A)** Hierarchical clustering of biotic stress-responsive EXO70 isoforms upon elicitor treatment. **(B)** Hierarchical clustering of EXO70 isoforms according to expression levels upon bacterial treatments. For gene terminology see **Figure 1**.

Further on, we focused on the selection of paralogs which were induced by various biotic stress treatments. There we could observe differential expression depending on the type of biotic stress. The expression patterns and hierarchical clustering of these isoforms, with EXO70A1 included as a reference for comparison, are shown for elicitor and bacterial treatments (**Figure 2**) and also for fungal and oomycete infection (**Supplementary Figure 2**, summarization in **Table 1**). Generally, the most responsive paralogs to elicitor and bacteria treatments are H1, H2, E2, B2, H4, H7, and B1, while to fungal and oomycete treatments B2, E2, and H1. Interestingly, the expression of EXO70B2 correlated with H4 and E1 in elicitor treatments, with B1 upon bacterial infection and with E1, B1, and E2 upon fungal and oomycete treatments. B1 and B2 also appear to correlate with F1 and H7.

**Table 1:** Summarization of biotic stress-related EXO70 paralog expression upregulation. Genes strongly upregulated (log2 ratio app. >2) in at least one study or experimental setup are shown in bold, moderately upregulated (log2 ratio app. < 2) in standard font.

Treatment		Upregulated clade members				
		EXO70A	EXO70B	EXO70E	EXO70F	EXO70H
**Elicitors**	chitaoctaose		**B1**, **B2**			**H1**, H2, H4, **H7**
	Elf18		**B2**	E1,**E2**		**H1**, **H2**, **H4**
	Flg22		B1, **B2**	E2	F1	**H1**, **H2**, **H4**, **H7**
	HrpZ		B1, **B2**	E1		**H1**, **H2**, H4
	oligogalacturans		B1, **B2**	E1		**H1**, **H2**, **H4**, H7
**Bacteria**	*Pseudomonas syringae*		B1, **B2**	E1, E2	F1	**H1**, **H2**, **H4**
**Non-host fungus**	*Blumeria graminis*		B1, B2	E1,**E2**		**H1**, H4, H7
**Host fungus**	*Alternaria brassicicola*		B1, **B2**	E1		**H1**, **H4**
	*Golovinomyces orontii*	A1	B1, **B2**	E1,**E2**		**H4**
	*Sclerotinia sclerotiorum*		B1, **B2**	E1,**E2**		H4, H7
**Oomycete**	*Hyaloperonospora arabidopsidis*		B1, **B2**	E1,**E2**		
	*Phytophthora parasitica*		**B1**, **B2**	**E1**	F1	**H1**, **H7**

Genes strongly upregulated (log2 ratio app. > 2) in at least one study or experimental setup are shown in bold, moderately upregulated (1 log2 ratio app. < 2) in standard font.

**Figure 3:**
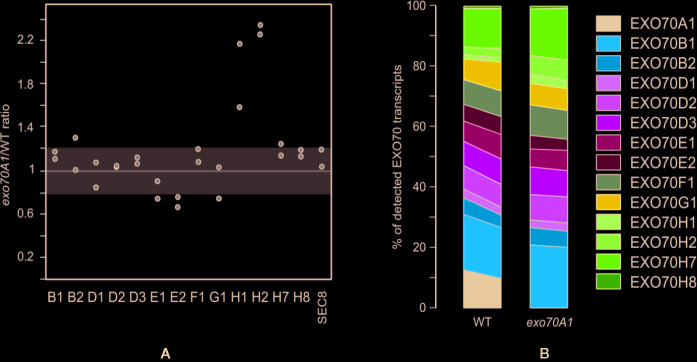
**(A)** Relative transcript levels of detectable EXO70 isoforms and the SEC8 subunit in *exo70A1* mutant *vs.* WT seedlings (from two replicates). Gray zone represents a less than 20% change in transcript abundance. **(B)** Composition of the EXO70 transcript population in *exo70A1* mutant *vs.* WT seedlings. Two replicates for each genotype are represented by the left and right sides of the corresponding stacked bar graph, respectively. For gene terminology see **Figure 1**.

In order to examine the ability of other EXO70 isoforms to compensate for loss of EXO70A1, we performed microarray analysis of gene expression in 7 days old light-grown *exo70A1* and WT seedlings using the Arabidopsis ATH1 chip (Hála et al., 2010 and Methods). In general, changes in expression levels of most EXO70 paralogs were rather modest (**Figure 3A**), but some differences were nevertheless observed. While EXO70B1 and H7 remained the most expressed paralogs in both WT and *exo70A1* seedlings, loss of EXO70A1 leads to further increase in relative transcript abundance of H-group paralogs, especially H1and H2, and to a lesser extent also of F1, as well as to a slight drop in relative abundance of E2 (**Figure 3B**). Even though the pollen specific isoform EXO70A2 mRNA levels were in our experiments under the detection limit, recent work by Markovic et al., 2020 indicates the importance of a sporophytic EXO70A2 function of in the *exo70A1* plants. Interestingly, the increase of H1 and H2 in both biotic stresses and in the absence of A1 implies that H1 and H2 might compensate functionally for loss of basal A1 level during pathogen response.

These analyses confirm that only some EXO70 isoforms have high expression levels under normal conditions and that the expression patterns mostly do not follow phylogenetic relatedness. Under the biotic stress conditions, expression of several paralogs from clade 2 is upregulated. Additionally, when the developmentally most important isoform EXO70A1 is missing, the compensatory upregulation is distributed among several clade 2 isoforms.

### Defense-Related Phenotypes of Selected Double Mutants

In order to further understand the involvement of multiple paralogs in biotic stress, we decided to perform the assay for seedling root hair growth stimulation by pathogenic bacteria (Pečenková et al., 2017a). For that purpose, we focused on intersection of paralogs expressed in root hairs (**Supplemental Figure 2**) and under the biotic stress (**Figure 2**), mainly EXO70B2, E1, F1, and H1, but also on the developmentally relevant EXO70A1 and EXO70B1. Besides single mutants of theses paralogs, we used also several combinations of double mutants (*exo70A1exo70B1, exo70A1exo70B2*, *exo70B1exo70B2, exo70B2exo70H1,* and *exo70E1exo70F1*) and one triple mutant *exo70A1exo70B2exo70H1* in order to detect the aggravation of defense-related phenotypes as an evidence of the mutual redundancy capacity. No obvious additive developmental or growth phenotypic deviations were observed in any of the double or triple mutant under standard culture conditions. All mutant combinations involving *exo70A1* resembled the previously characterized single *exo70A1* dwarf mutant (Synek et al., 2006), while any mutant carrying *exo70B1* exhibited leaf HR-like lesions as previously reported for the single *exo70B1* and *exo70A1exo70B*1 mutants (Kulich et al., 2013).

**Figure 4:**
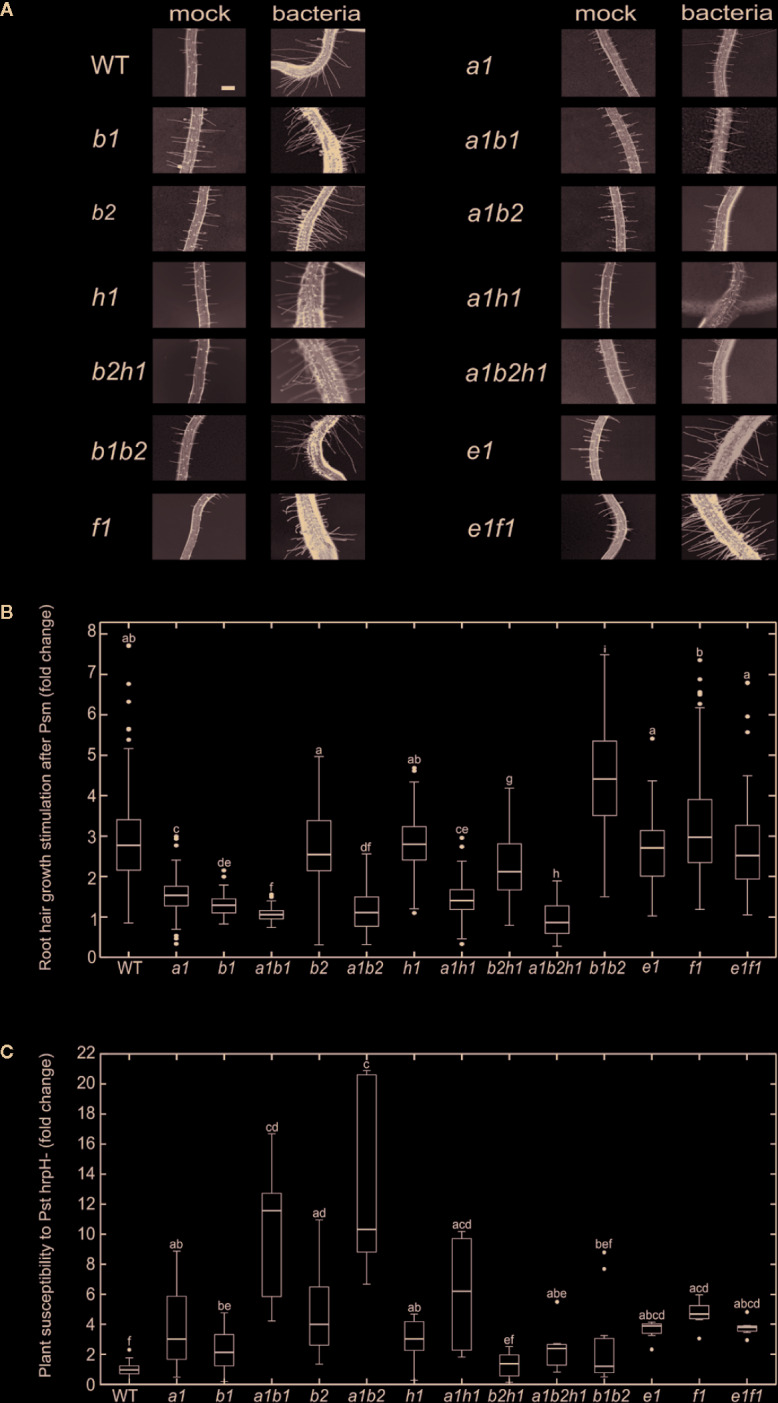
**(A)** Root hair growth appearance 48 h after mock or *P. syringae* pv. *maculicola* (Psm) treatment for each of analyzed lines (bar = 100 µm). **(B)** Root hair growth stimulation after *P. syringae* pv. *maculicola* (Psm) inoculation. For each of mutant lines root hair length is compared to mock treated control and the difference expressed as a fold change; the mock treated WT root hair size represents fold change 1; error bars—standard deviation, n = 60–407 root hairs; different letters indicate significant differences (P < 0.05). **(C)** *P. syringae* pv. *tomato* DC3000 *hrpH−* flooding inoculation. The variation in sensitivity is expressed as a fold change of CFU in comparison to the WT (fold change 1) and is presented for each of analyzed lines; error bars—standard deviation, n = 6–36 samples from three to four pooled seedlings each; different letters indicate significant differences (P < 0.05).

We first looked for possible effects of these mutations on the responses elicited by biotic stress by quantifying root hair growth stimulation in single and double mutants after application of pathogenic bacteria *Pseudomonas syringae* pv. *maculicola* (Psm) to root tips (Pečenková et al., 2017a). Root hair response to bacterial challenge involves the perception of bacteria presence, response to pathogenic bacteria, and root hair elongation by tip growth, *i.e.* processes involving the exocyst and possibly specific EXO70 isoforms. Most of the analyzed single mutants exhibited significantly smaller stimulation of root hair growth than WT plants, most prominently double and triple mutants comprising *exo70A1* (**Figures 4A, B**; root hair appearance of selected lines in **Supplemental Figure 3**). Some of the analyzed mutant lines exhibit shorter or longer root hairs than WT plants already in conditions of mock treatment (**Supplementary Figure 3**). To eliminate these effects when focusing specifically on the biotic stress responses, we evaluated the effects of biotic stress by measuring the ratio of bacteria- *versus* mock-treated root hair lengths. Interestingly, in the case of *exoB1exo70B2* mutants we observed the opposite and counterintuitive effect, *i.e.* enhanced root hair growth after bacterial stimulation when compared to the single mutants’ responses.

In order to further follow pathogen perception-related defects, we also examined the same set of mutants for their ability to sustain propagation of *Pseudomonas syringae* pv. *tomato* (Pst) after flooding inoculation (Ishiga et al., 2011). A nonvirulent Pst hrpH-strain was used, avoiding thus effector-responsive component of plant defense. All of the tested mutant lines were normalized to their corresponding wild type, allowing thus a comparison of the relative strength of response among different mutants (**Figure 4C**). Again, most of the analyzed mutant lines have significantly compromised resistance toward Pst hrpH−. Surprisingly and contrasting to the situation with single mutants, double mutant combinations *exoB1exo70B2* and *exo70B2exo70H1* seem to sustain the bacterial inoculation to a level comparable to WT. Interestingly, combinations of some mutants (*exo70B1*, *exo70B2* and *exo70H1*) with *exo70A1* increase the susceptibility toward hrpH−, but the triple mutant *exo70A1exo70B2exo70H1* has again sensitivity “corrected” to single *exo70A1* mutant levels.

These results evidence a more complex pattern of functional overlap that includes also the compensatory effect among EXO70 mutations, probably as a consequence of competition of isoforms for the core of the exocyst complex.

### Diversity and Preferences of EXO70 Lipid-Binding Domains

**Figure 5:**
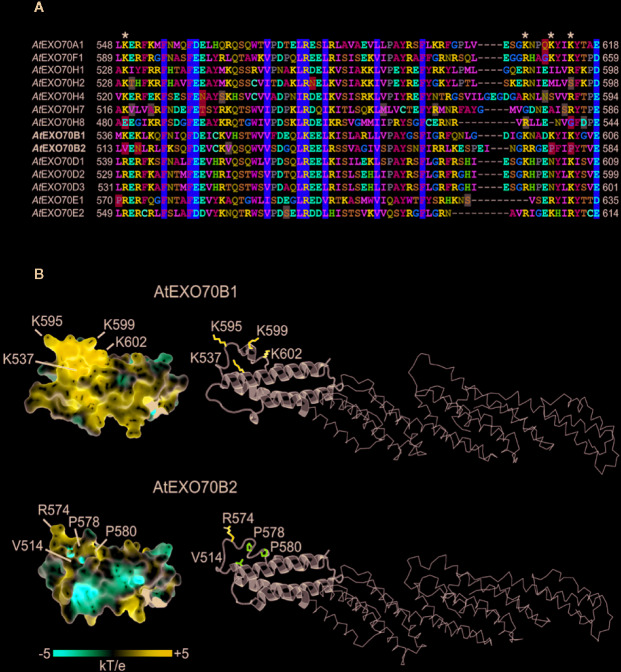
Alignment of predicted lipid-interacting motifs of selected EXO70 paralogs **(A)** and 3D structures of C termini of EXO70B1 and EXO70B2 **(B)**. Residues shown on yellow background are conserved in all isoforms; positions with basic and acidic residues were found to be conserved as well (blue and red letters, respectively); a pair of prolins and valine replacing basic amino acids in the putative binding motif was found in EXO70B2 but not in EXO70B1; several conserved glutamate/aspartate residues are replaced by an asparagine residue in some of EXO70H isoforms (blue boxes; **A)**. The visualization of electrostatic potential of EXO70B1 C-terminal domain revealed positively charged pocket containing three lysine residues that are mutated in EXO70B2, resulting in significant loss of positive charge **(B)**.

To explore the possibility that the phospholipid-binding capacity of EXO70 isoforms C-termini could be a determinant of localization and therefore also functional specificity, we compared the amino acid sequences of the EXO70 protein C-termini containing a predicted membrane-binding site (Žárský et al., 2009; Cvrčková et al., 2012). Several highly conserved motifs among all paralogs were detected (yellow boxes in **Figure 5A**); besides, stretches of basic and acidic residues were found to be conserved as well (blue and red letters, respectively). Combining the data from gene expression and lipid-binding *in silico* analysis, we could pinpoint several candidates for mutations that might have resulted in within-clade diversification of membrane-binding specificities. We focused in particular on EXO70B1 and B2 since they behaved in different manner in our biotic response assays. Indeed, a pair of prolines and a valine replacing basic amino acids in the putative phospholipid-binding motif were found in EXO70B2 but not in EXO70B1; one of the basic amino acid is also not conserved in the H group isoforms. Furthermore, several conserved glutamate/aspartate residues are replaced by an asparagine residue in some of EXO70H isoforms (**Figure 5A**).

In order to further predict a functional role of the amino acid residues that are mutated in EXO70B2 C-terminus, we modeled 3D structures of EXO70B1 and B2 using known EXO70 structures as templates. Importantly, the visualization of electrostatic potential of EXO70B1 C-terminal domain revealed a positively charged pocket containing three lysine residues that are mutated in EXO70B2. While the overall backbone structures of EXO70B1 and B2 are very similar and there is only a small structural difference between predicted EXO70B1 and B2 C-termini (RMSD = 0.306), the three lysines mutated to prolines and valine in EXO70B2 result in significant loss of positive charge (**Figure 5B**). Moreover, the presence of the two other backbone-bending mutations ((L > P and K > P) affects the shape of EXO70B2 C terminus near the putative membrane-binding pocket (**Figure 5B**).

**Figure 6:**
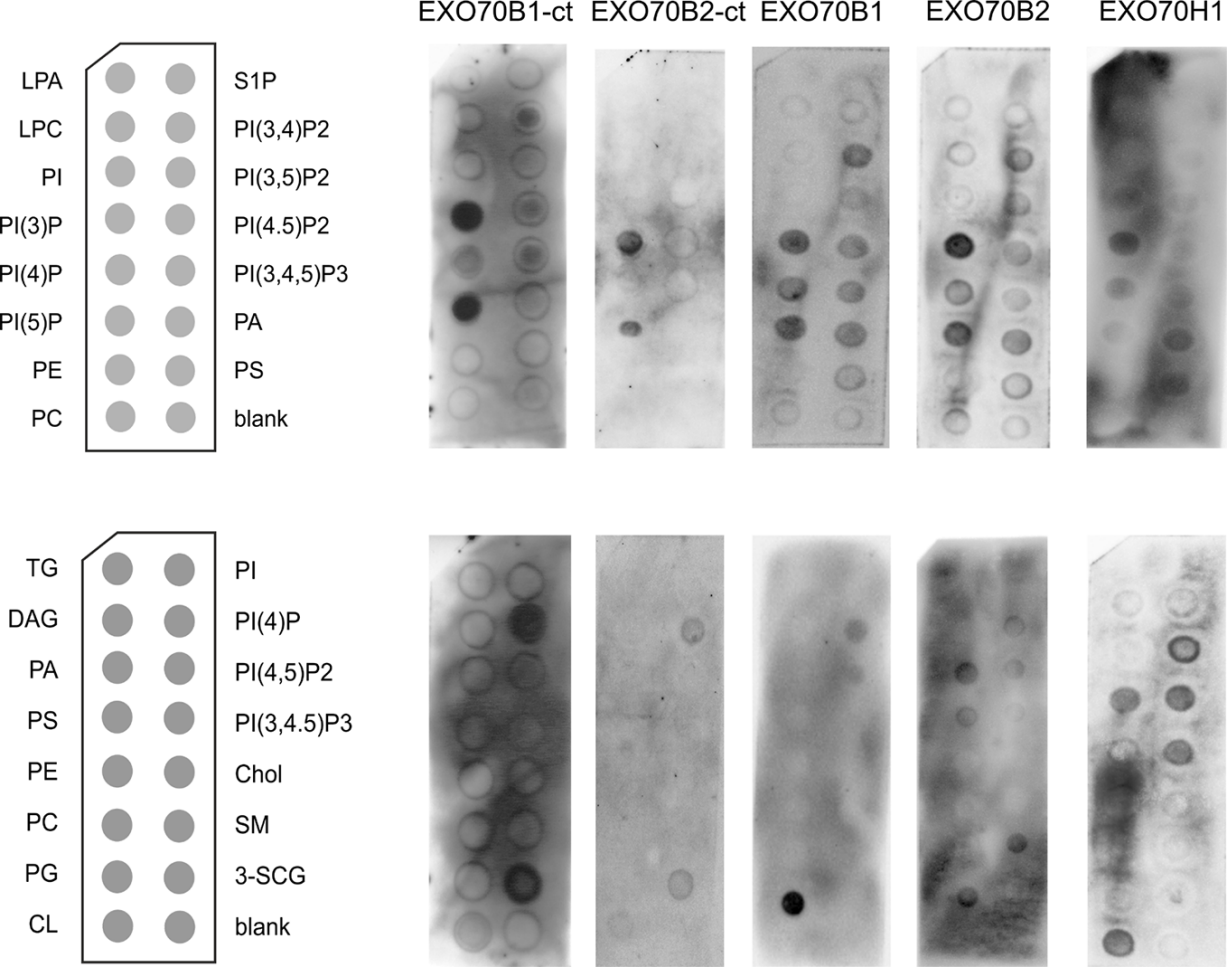
Phosphoinositide-(upper row) and membrane lipid-binding capacity (lower row) of EXO70B1 and EXO70B2 C-terminal fragments (-ct; left) and full length variants (right) of EXO70B1, B2, and H1. For lipid terminology see *Abbreviations*.

We further assessed how the differences found between the EXO70B1 and B2 C-termini will affect their lipid-binding capacity using an *in vitro* assay. For this purpose, we used qualitative *in vitro* assays of lipid binding profiles employing PIP and membrane lipid strips (thus, the results might not fully correspond to the situation *in vivo*). The C-terminal fragments of EXO70B1 and B2 (aa 431-624 and 415-599, respectively) were expressed in *E. coli* as GST fusions (**Supplementary Figure 4A**). Purified fusion proteins were incubated with different phosphatidylinositol phosphates (PIP) immobilized as spots on membrane strips (**Figure 6**, upper row). Interestingly, despite their amino acid differences in lipid-binding domain, the two EXO70B isoforms appear to bind a similar repertoire of PIP species—most strongly PI(3)P and PI(5)P monophosphates. The PIP-strip assays were also performed with full-length HA-tagged EXO70B1 and B2 proteins produced in an *in vitro* translation system, together with the less related isoform EXO70H1 (**Figure 6**, upper row, on the right; **Supplementary Figure 4B**). Similar lipid-binding affinity for all analyzed isoforms was also detected using membrane lipid strips (**Figure 6**, lower row). The full length versions of the tested EXO70s were found to interact weakly also with phosphatidylserine (PS) and phosphatidic acid (PA), unlike their C-terminal fragments, indicating the presence of protein regions outside the C-terminal motif contributing to lipid-binding specificity. Nevertheless, the analyzed proteins do not bind to lipids on strips with the same intensity (*e.g.* for EXO70B1 positive spots are much fainter than in the case of EXO70B2 under the same exposure time; **Supplemental Figure 4B**), at least partially as a consequence of different efficiencies of *in vitro* translation, but possibly also of intrinsic lipid-binding affinities, a matter requesting further alternative quantitative assays.

Despite their diverged lipid-binding domains and distinct roles in the plant cell context, the lipid-binding analysis shows that the EXO70B1 and B2 isoforms could bind the same type of lipids, only slightly different from more distantly related EXO70H1. This suggests that their sequence diversification affects other types of interactions, probably protein–protein, that might result in functional specialization.

## Discussion

In this work, we aimed to map and assess some aspects of functional overlap (redundancy) and specialization among Arabidopsis EXO70 isoforms. We are building on the working hypothesis that redundancy can be expected among those paralogs that (i) exhibit the closest evolutionary relationship, (ii) have overlapping expression patterns and lipid affinities, and that (iii) combination of their multiple loss-of-function mutations leads to more severe phenotypic manifestations compared to single mutations.

Similarly to other rapidly diversifying gene families, the expression pattern of *A. thaliana* EXO70 isoforms does not mirror their mutual relationships inferred by phylogenetic analyses (compare *e.g.* Dvořáková et al., 2007). Also isoforms with similar expression patterns may have unique roles; for instance, the two paralogs with the highest expression throughout the Arabidopsis development are EXO70A1 and EXO70B1, documented to function differently—while EXO70A1 plays a part in the exocytosis related to the polar growth, cytokinesis, cell expansion, and endosomal recycling, B1 engages in the autophagic route to the vacuole (Kulich et al., 2013). The *exo70A1* mutant has pleiotropic developmental defects, including very obvious stunted growth of both roots and rosettes (Synek et al., 2006), while *exo70B1* plants first develop normally; however, in adult rosettes, they develop spontaneous hypersensitive response (HR)-like leaf lesions, display partial loss of anthocyanin vacuolar accumulation, and are more resistant to biotrophic and hemi-biotrophic pathogens due to elevated salicylic acid (SA) levels in fully mature plants (Kulich et al., 2013; Zhao et al., 2015). HR- and SA-related *exo70B1* phenotypes can be suppressed by mutations in the R-related protein TIR-NBS2 [TN2 (Zhao et al., 2015)]. Considering that the two isoforms have high overall sequence similarity (including the C-terminal membrane-binding motif), it is possible to speculate that additional isoform-specific protein interactors are instrumental for the observed differences—*e.g.* the known defense-related protein RIN4 has been shown to specifically interact with EXO70B1 but not with EXO70A1 (Sabol et al., 2017).

Surprisingly, according to our bacterial flooding assays, EXO70A1 loss-of-function has significant impact on plant defense capability, a finding never reported before, mainly due to the expectation that the biotic-stress induced isoforms play more important role in plant defense. Nevertheless, it is obvious that EXO70A1 has an important general background role in defense as well, probably due to its influence on biogenesis and maintenance of plasma membrane and especially on cell wall biogenesis and properties (Fendrych et al., 2013; Vukašinović et al., 2017). Surprisingly, unlike some other dwarf and esp. *atg* mutants (Zhang et al., 2007; van Wersch et al., 2016) and also unlike other exocyst mutants such as *exo70B1* and *Ossec3A* (Kulich et al., 2013; Ma et al., 2018), *exo70A1* shows no elevation of either salicylic acid (SA) biosynthetic machinery or in overall SA content (Žárský et al., in preparation). Based on all of this, we could speculate that the increased susceptibility in the case of double mutant *exo70A1 exo70B1*, which otherwise has the same developmental phenotype as the single *exo70A1* mutant, is rather a cumulative effect of the two mutations converging in the situation of biotic stress than the aggravation of the single mutants’ phenotypes.

The second Arabidopsis EXO70B isoform, EXO70B2, is important for efficient defense against pathogens (Pecenková et al., 2011; Stegmann et al., 2012; Stegmann et al., 2014; Du et al., 2015; Du et al., 2018);. EXO70A1 and B2 have different expression patterns and highly diversified lipid binding domains. The B1 and B2 isoforms are closely related and interact with both common and specific protein partners, some of them important components of the pathogen-associated molecular patterns-triggered immunity (PTI) and effector-triggered susceptibility and immunity pathways [ETS and ETI; see (Jones and Dangl, 2006)]. Interestingly in most Angiosperm families such diversification of EXO70B functions did not happen, and only a single EXO70B isoform is present (Cvrčková et al., 2012). Recently co-operation of the Arabidopsis EXO70B1 and B2 isoforms in the regulation of FLS2 flagellin receptor kinase plasma membrane localization was described (Wang et al., 2020). Despite the overlaps in expression and lipid-binding capacity, there may be a compensatory effect between the two mutations (Drs et al., unpublished observation). It is possible that the clue for this unexpected outcome lies within the EXO70Bs different protein interacting repertoire (Zhao et al., 2015; Sabol et al., 2017).

Similarly to the double mutants *exo70A1exo70B1*, and in conflict with the *exo70B1exo70B2* phenotype case, *exo70A1exo70B2* appears to be more severely compromised in the defense responses analyzed in the present work compared to the single mutant counterparts, similarly to what has been found in the *exo70A1exo70B2* defense reaction to *Blumeria graminis* (Žárský et al., 2013). Unlike the case of the double mutant *exo70A1exo70B1* with secretory impairment caused by *exo70A1* and additional defect in autophagy/vacuolar transport imposed by *exo70B1* (Kulich et al., 2013), the double mutant *exo70A1exo70B2* severe phenotype may be the consequence of the lack of partial redundancy between the EXO70A1-related constitutive secretion and EXO70B2-related defense secretion.

The complexity of relationships among EXO70s is well-illustrated by the example of root hair growth enhancement in *exo70B1exo70B2* mutants, consistent with the possible competition of the EXO70B clade paralogs and the tip growth-promoting EXO70A1 paralog for exocyst core subunits. Neither the of two EXO70Bs isoforms has prominent affinity for phosphatidyl serine (PS), a tip-growth localized lipid in root hairs (Platre et al., 2018). We could thus speculate that in the absence of both EXO70Bs, the whole pool of exocyst core subunits in root hair cells is available for tip-growth-related and preferentially PS-binding EXO70 isoform(s), possibly the most abundant EXO70A1 (Fendrych et al., 2013). The ability of EXO70B1 and B2 to bind PI(3)P and PI(5)P suggests their possible involvement in processes not directly related to cell enlargement. Although PI(3)P is a minor lipid in the cell, it is a key player in membrane protein recruitment, especially late endosomal vesicle trafficking and autophagosome formation (Balla, 2013; van Wersch et al., 2016). This is in concordance with the autophagy-related function of EXO70B1 (Kulich et al., 2013), and it remains to be seen if and how the EXO70B2 could be as well involved in autophagy.

The EXO70E1, F1 and H1 are probably the least studied Arabidopsis EXO70 paralogs. Our observation of compensatory E1/F1 and B2/H1 (also A1/B2/H1) effects thus may be opening a new field that would deserve attention in the nearest future.

In our pathogen sensitivity assays, we could often see a complementarity of responses between the root hair growth and general defense. The single mutant lines that did not respond to root hair growth enhancement to the same level as the WT were also found to be more sensitive in the overall immunity to *P. syringae* in flooding assays. Also the double mutants that had diminished capacity to enhance root hair growth after bacteria inoculation were more sensitive to bacteria in flooding experiments (*e.g. exo70A1exo70B1* and *exo70A1exo70B2*) in comparison to corresponding single mutants. This strengthens a suitability of the root hair growth response assay as a readout of the general defense capacity. On the other hand, the case of the triple mutant *exo70A1exo70B2exo70H1* fully reflects the complexity of relationships among isoforms—even though it is the mutant with the most prominent reduction of root hair growth (possibly also due to a general cell expansion defect), it is not the one to be the most sensitive toward bacteria in flooding assay.

Our analysis of lipid-interacting motifs suggests that Arabidopsis EXO70 isoforms might have multiple lipid interaction specificities, some of them located outside the C-terminal motif predicted to be the a major specificity determinant based on animal and yeast PI(4,5)P_2_-binding homologs, similarly to what has been suggested for yeast Exo70p (Pleskot et al., 2015). The EXO70 lipid binding specificity can also be modulated by additional protein interactions, including putative EXO70s homo- or hetero-dimerization, which could help a particular isoform to achieve a highly specific function, that may even be independent from the rest of the complex, as shown for membrane curvature in animal cells (Zhao et al., 2013) or EXO70C2 in pollen tubes (Synek et al., 2017). Although the EXO70B2 isoform can homodimerize in the yeast two hybrid assay (Pecenková et al., 2011), biological relevance of plant EXO70 dimerization capability *in vivo* remains elusive (Žárský et al., 2020) and only recently was implied in the FLS2 plasma membrane localization (Wang et al., 2020).

To conclude, our results demonstrate that despite evolutionary relatedness, overlapping mRNA expression patterns, and similar lipid binding affinities, each EXO70 isoform might have a unique function. When one isoform is missing, there is mostly no simple full replacement of one isoform function by another one. Our data indicate that the pool of exocyst core subunits is possibly available for several EXO70 isoforms present in the same cell so that the overall endomembrane dynamics within the cell is also regulated in different situations by the competition of different EXO70s for the same core subunits (Žárský et al., 2013). The main question of other isoforms’ involvement in secretion *versus* autophagy will necessitate creation of additional multiple mutants involving other paralogs (especially H1 and H2) and further analysis of multiple mutants’ phenotypes beyond defense-related phenomena.

## Data Availability Statement

Publicly available datasets were analyzed in this study. This data can be found here: GSE18986.

## Author Contributions

TP performed root hair assays, RNA expression analysis, mutant crossing, recombinant protein expression, lipid-binding experiments and wrote the manuscript. AP performed Pseudomonas flooding experiments. MP conceptualized and performed lipid-binding domain *in silico* analysis and statistical analysis of biotic assay results. JO performed mutant propagation, protein expression *in vitro*, crossings, and root hair analysis. MD performed mutant propagation, image analysis, and text editing. EJ and LS performed RNA microarray experiments and data analysis. PP performed cloning. HS performed mutant propagation and crossings. VŽ performed planning of experiments, interpretation and writing. FC performed data analysis, figure design, and participated in data interpretation and manuscript writing. All authors contributed to the article and approved the submitted version.

## Funding

This research was funded by the Ministry of Education, Youth and Sports of the Czech Republic (MŠMT) project and European Regional Development Fund project CZ.02.1.01/0.0/0.0/16_019/0000738 “Centre for Experimental Plant Biology” at the Institute of Experimental Botany and by the Czech Science Foundation (GAČR) grants number CSF 19-02242J and GC18-18290J.

## Conflict of Interest

The authors declare that the research was conducted in the absence of any commercial or financial relationships that could be construed as a potential conflict of interest.

## Abbreviations

3-SGC, 3-sulfogalactosylceramide; Chol, Cholesterol; CL, Cardiolipin; DAG, Diacylglycerol; dH_2_O, Distilled water; EXO70 Exocyst subunit 70 (named according to its ***ca.*** 70 kDa size); HR, Hypersensitive response; LOF, Loss of function; LPA, Lysophosphatidic acid; LPC, Lysophosphocholine; PA, Phosphatidic acid; PC, Phosphatidylcholine; PE, Phosphatidylethanolamine; PG, Phosphatidylglycerol; PI, Phosphatidylinositol; PI(3)P, Phosphatidylinositol (3) phosphate; PI(3,4)P2, Phosphatidylinositol (3,4) bisphosphate; PI(3,5)P2, Phosphatidylinositol (3,5) bisphosphate; PI(3,4,5)P3, Phosphatidylinositol (3,4,5) trisphosphate; PI(4)P, Phosphatidylinositol (4) phosphate; PI(4,5)P2, Phosphatidylinositol (4,5) bisphosphate; PI(5)P, Phosphatidylinositol (5) phosphate; PIP, Phosphoinositides; PS, Phosphatidylserine; S1P, Sphingosine 1-Phosphate; SA, Salicylic acid; SM, Sphingomyelin; TG, Triglyceride; WT, Wild type.
