# Supplementary material for: Redundant and Diversified Roles Among Selected Arabidopsis thaliana EXO70 Paralogs During Biotic Stress Responses
Source: Front Plant Sci. 2020 Jun 26;11:960. doi: 10.3389/fpls.2020.00960 (PMC7333677; doi:10.3389/fpls.2020.00960)
Supplement: Supplementary file 1 [file DataSheet_1.pdf]

Pečenková *et al*.: Redundant and diversified roles among selected EXO70 paralogs of *Arabidopsis thaliana* during biotic stress responses

Supplementary materials

Supplementary figure 1. Hierarchical clustering of EXO70 isoforms according to expression levels across complete set of all cell types and anatomical parts covered in the Genevestigator database.

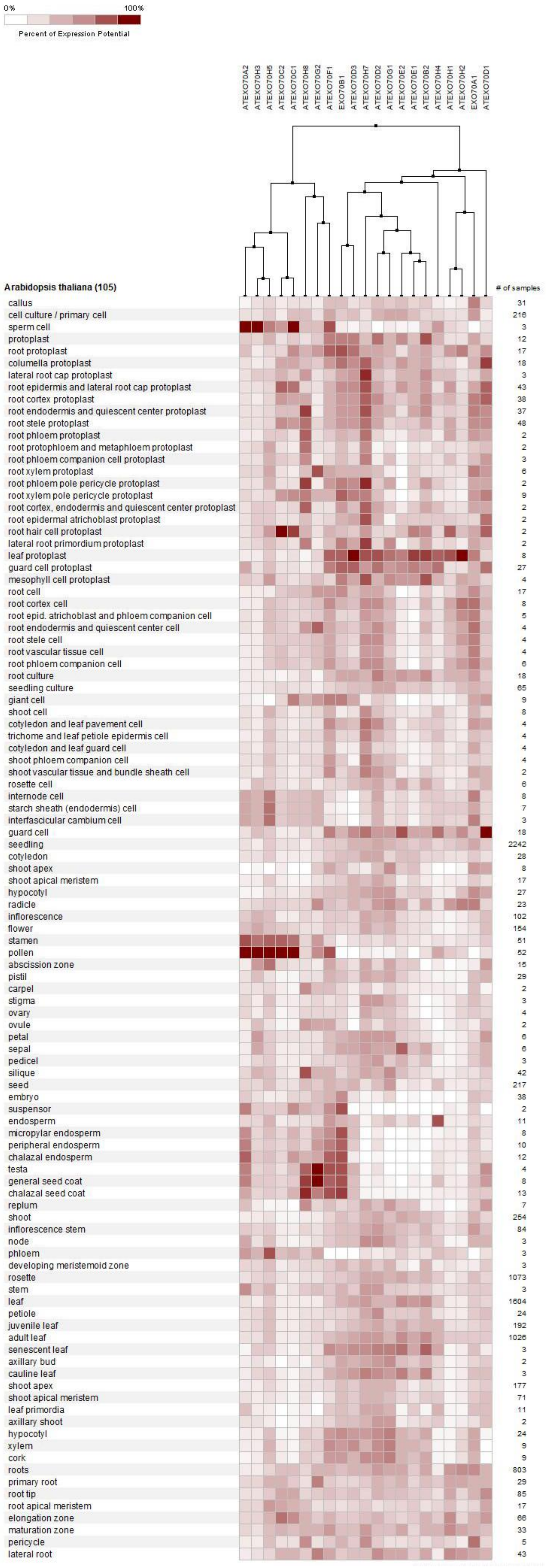

Supplementary figure 2. Hierarchical clustering of EXO70 isoforms according to expression levels upon fungal and oomycete treatments.

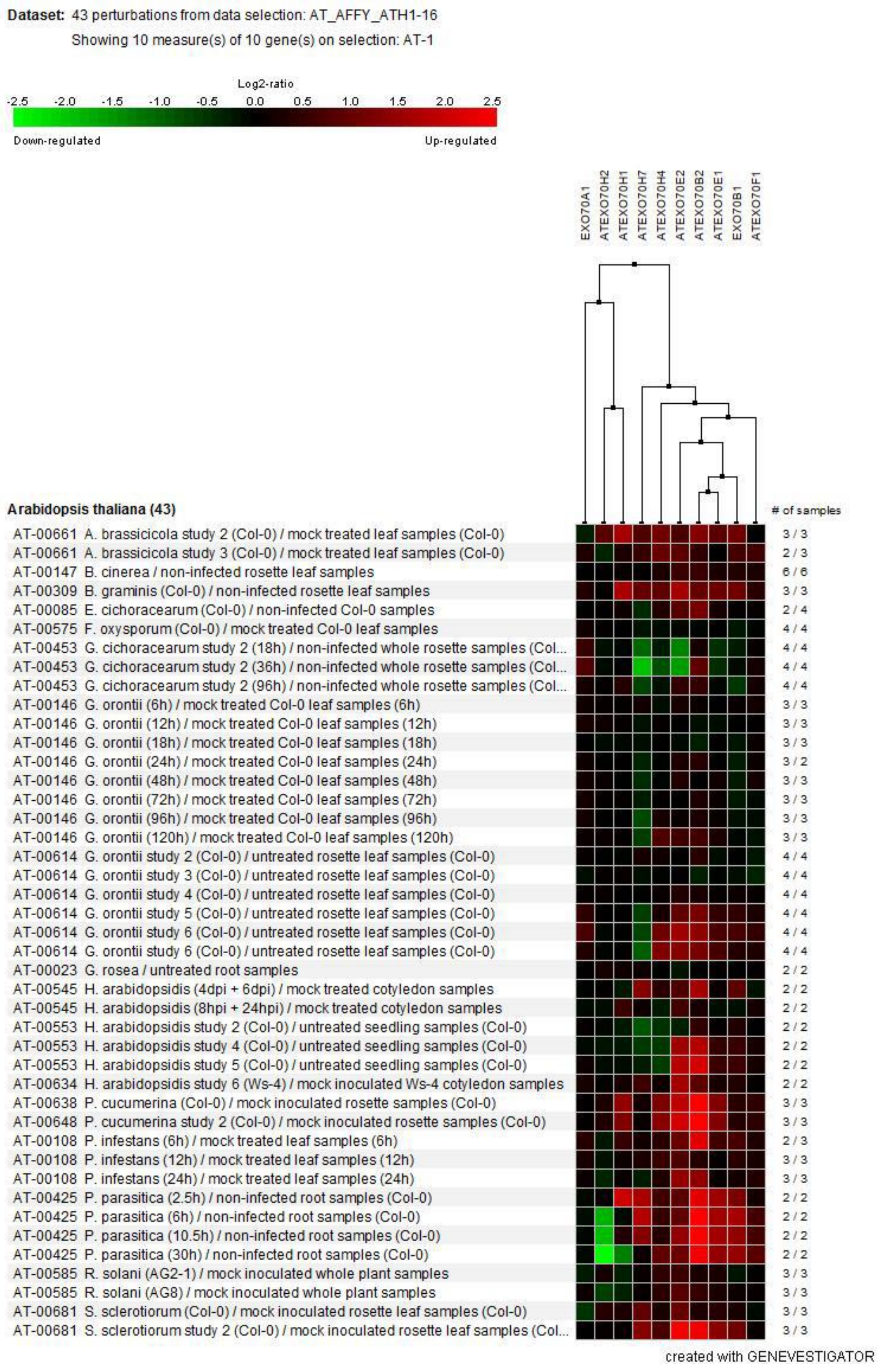

Supplementary figure 3. Absolute root hair lengths of mock treated seedlings for analyzed single and multiple mutant lines.

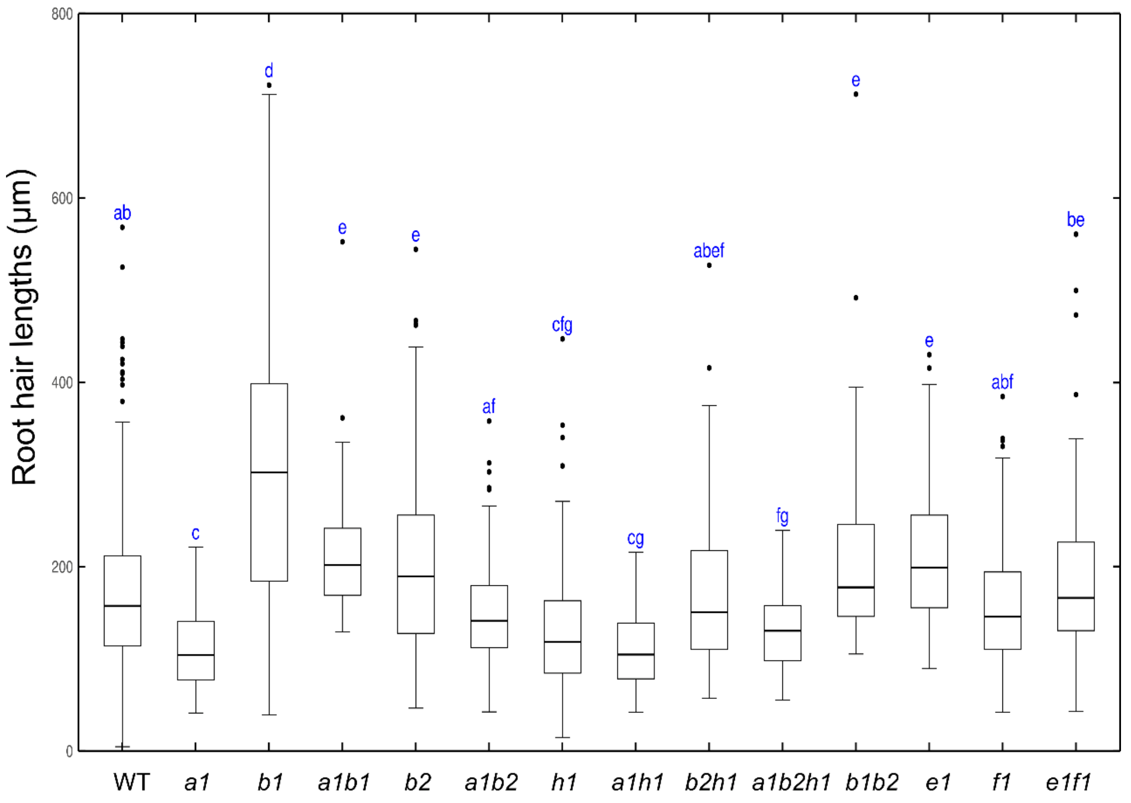

Supplementary figure 4. (A) Coomassie stained gels with aliquots of GST-fused EXO70B1 and B2 C termini as well as free GST (upper row); PIP/GST as negative and PIP/GRIP protein (Echelon, Israel) as positive strip controls (lower row). (B) Ponceau stained membranes and anti-HA (Sigma) detection of *in vitro* translated HA-tagged EXO70B1, B2 and H1 (4x more amount used for incubation with strips); comparison of the strength of signal detected on PIP strips for EXO70B1 and B2 when equal amount of proteins and exposure times for signal detection were used.

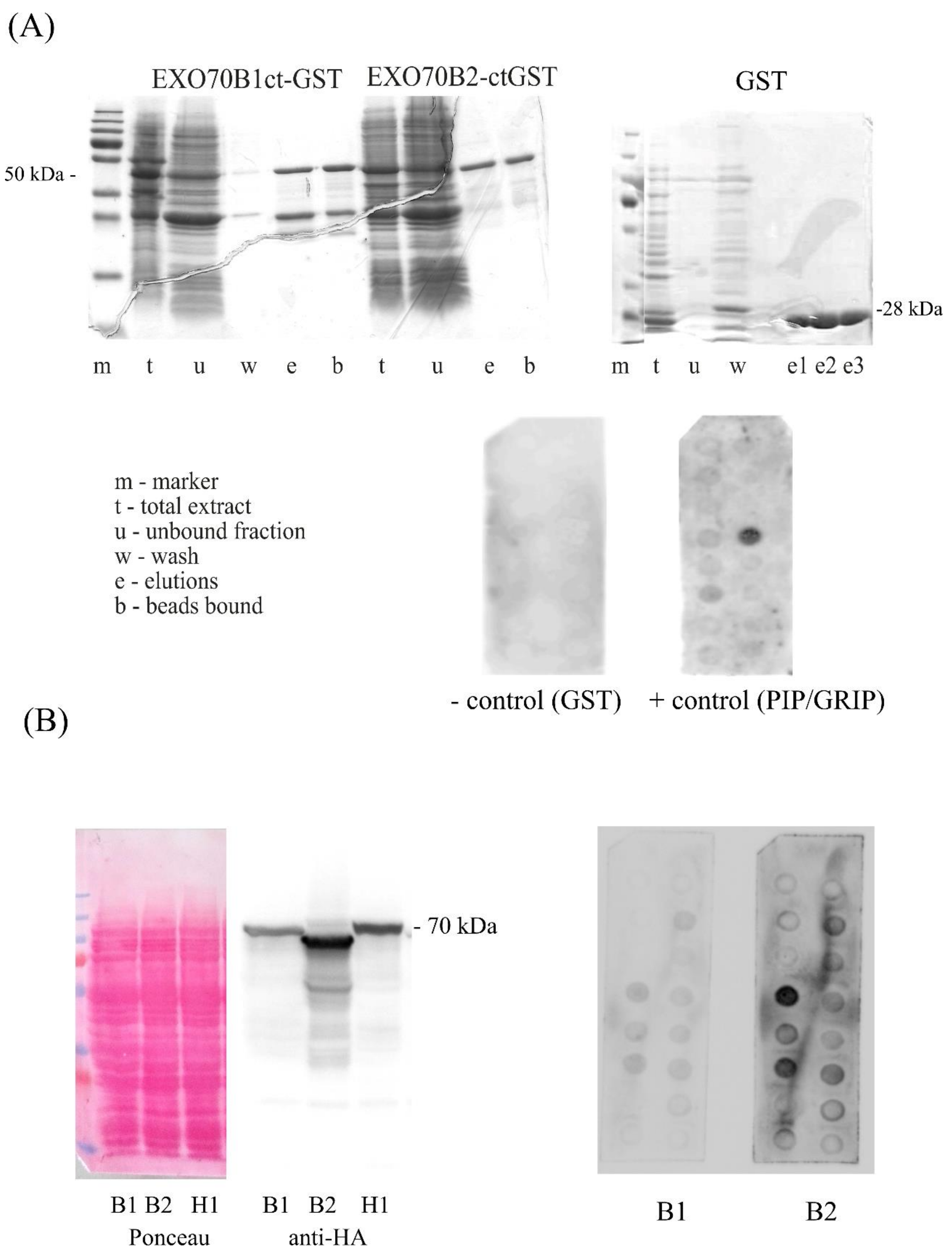

Supplementary table 1. Primers used for cloning and genotyping.

Purpose	Forward primer[1]	Reverse primer[1]
GST-EXO70B1-ct (EcoRI-SalI)	aagaattctctaaagattcaactctgttgactg	aaggtcgactcattttgttcccgtgg
GST-EXO70B2-ct (EcoRI-XhoI)	aagaattcggatcagatacgagaccgttg	aaactcgagtcaacttgagctttcctt
HA-EXO70B1	gatgttccagattacgctatggcggagaatggtg	atagcggccgctcattttcttcccgtggt
HA-EXO70B2	gatgttccagattacgctatggctgaagccggt	atagcggccgctcaacttgagctttccttg
HA-EXO70H1	gatgttccagattacgctatggcgaaaatggcga	atagcggccgctcagcctgaaacacacc
pTNT-HA-overhang_SalI[2]	aagtcgacgccgccaccatgtacccatacgatgttccagattacgctatg	
pTNT-HA-overhang_XhoI[3]	atactcgaggccgccaccatgtacccatacgatgttccagattacgctatg	
exo70E1	ctgatgatgttcttcaaatagctatgg	acagaggttatggagtagaaatgtcc

[1] All primer sequences are presented in 5′to 3′ direction. Introduced restriction sites are underlined.

[2]Used for B1,H1 SalI-NotI cloning. [3]Used for B2 XhoI-NotI cloning.

Supplementary table 2. Sample sizes (n) used for analyzes in biotic stress assays. For mock and bacteria treated root hairs (RH) total numbers of root hairs and in parenthesis numbers of seedlings analyzed are presented (ca 20 root hairs per seedling). For flooding experiments numbers of analyzed samples (each sample is pool of 3-4 seedlings) are presented.

	**n for mock treated RH**	**n for bacteria treated RH**	**n for hrpH- flooding**
**WT**	407 (20)	303 (15)	36
a1	120 (6)	160 (8)	18
b1	80 (4)	80 (4)	18
a1b1	80 (4)	80 (4)	12
b2	160 (8)	160 (8)	18
a1b2	123 (6)	103 (5)	12
h1	120 (6)	100 (5)	18
a1h1	60 (3)	120 (6)	12
b2h1	80 (4)	80 (4)	12
a1b2h1	80 (4)	80 (4)	10
b1b2	100 (5)	100 (5)	12
e1	135 (7)	116 (6)	6
f1	152 (5)	119 (6)	6
e1f1	120 (6)	120 (6)	6
